# Characterization of the mechanism by which a nonsense variant in *RYR2* leads to disordered calcium handling

**DOI:** 10.14814/phy2.15265

**Published:** 2022-04-19

**Authors:** Claire Hopton, Anke J. Tijsen, Leonid Maizels, Gil Arbel, Amira Gepstein, Nicola Bates, Benjamin Brown, Irit Huber, Susan J. Kimber, William G. Newman, Luigi Venetucci, Lior Gepstein

**Affiliations:** ^1^ Division of Evolution and Genomic Sciences Faculty of Biology, Medicine and Health University of Manchester Manchester UK; ^2^ Manchester Centre for Genomic Medicine Manchester University NHS Foundation Trust Health Innovation Manchester Manchester UK; ^3^ Division of Cardiovascular Sciences Faculty of Biology, Medicine and Health University of Manchester Manchester UK; ^4^ The Rappaport Faculty of Medicine and Research Institute Technion‐Institute of Technology Haifa Israel; ^5^ Amsterdam UMC Department of Experimental Cardiology Amsterdam Cardiovascular Sciences University of Amsterdam Amsterdam The Netherlands; ^6^ Division of Cardiology Sheba Medical Center Hospital Tel Hashomer Israel; ^7^ The Sackler Faculty of Medicine Tel Aviv University Tel Aviv Israel; ^8^ The Talpiot Sheba Medical Leadership Program Israel; ^9^ Division of Cell Matrix Biology and Regenerative Medicine Faculty of Biology, Medicine and Health University of Manchester Manchester UK; ^10^ Department of Cardiology Wythenshawe Hospital Manchester University NHS Foundation Trust Manchester UK; ^11^ Manchester Heart Centre Manchester University NHS Foundation Trust Health Innovation Manchester Manchester UK; ^12^ Cardiology Department Rambam Health Care Campus Haifa Israel

**Keywords:** CPVT, human induced pluripotent stem cells, RYR2, ventricular arrhythmia

## Abstract

Heterozygous missense variants of the cardiac ryanodine receptor gene (*RYR2*) cause catecholaminergic polymorphic ventricular tachycardia (CPVT). These missense variants of *RYR2* result in a gain of function of the ryanodine receptors, characterized by increased sensitivity to activation by calcium that results in an increased propensity to develop calcium waves and delayed afterdepolarizations. We have recently detected a nonsense variant in *RYR2* in a young patient who suffered an unexplained cardiac arrest. To understand the mechanism by which this variant in *RYR2*, p.(Arg4790Ter), leads to ventricular arrhythmias, human induced pluripotent stem cells (hiPSCs) harboring the novel nonsense variant in *RYR2* were generated and differentiated into cardiomyocytes (RYR2‐hiPSC‐CMs) and molecular and calcium handling properties were studied. RYR2‐hiPSC‐CMs displayed significant calcium handling abnormalities at baseline and following treatment with isoproterenol. Treatment with carvedilol and nebivolol resulted in a significant reduction in calcium handling abnormalities in the RYR2‐hiPSC‐CMs. Expression of the mutant *RYR2* allele was confirmed at the mRNA level and partial silencing of the mutant allele resulted in a reduction in calcium handling abnormalities at baseline. The nonsense variant behaves similarly to other gain of function variants in *RYR2*. Carvedilol and nebivolol may be suitable treatments for patients with gain of function *RYR2* variants.

## Graphical abstract

The *RYR2* gene encodes for the cardiac ryanodine receptor, a major calcium handling channel in cardiomyocytes. Heterozygous variants within the *RYR2* gene cause the inherited arrhythmia syndrome catecholaminergic polymorphic ventricular tachycardia (CPVT). The majority of these variants are missense changes and lead to a phenotype by a gain of function mechanism. We identified a novel nonsense variant in *RYR2* in young women who suffered a cardiac arrest and show, using cardiomyocytes derived from induced pluripotent stem cells, that this variant appears to lead to abnormal calcium handling in a similar mechanism to the previously described gain of function variants.

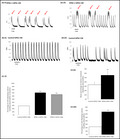

## 1 INTRODUCTION

The *RYR2* gene encodes for the cardiac ryanodine receptor, a major calcium handling channel located within cardiomyocytes. Most disease‐causing variants in *RYR2* are missense variants (Olubando et al., 2020; van der Werf et al., 2012) and result in an autosomal dominant form of catecholaminergic polymorphic ventricular tachycardia (CPVT1, MIM 604772). CPVT is characterized by adrenergically mediated arrhythmias, typically bidirectional or polymorphic VT, in the presence of a structurally normal heart (Leenhardt et al., 1995). The majority of these disease‐causing variants are clustered within four regions of *RYR2* (Supplementary Data, Figure S1). Most *RYR2* variants which have been functionally studied have been shown to result in a gain of function of RYR2, causing increased sensitivity of the channel to activation by calcium and an increased open probability (Jiang et al., 2004, 2005). Despite this, a small number of missense variants in *RYR2* have been shown to result in a loss of function and possibly cause arrhythmias by a different mechanism however further work is needed to fully understand these mechanisms (Zhao et al., 2015). Determining the mechanisms by which *RYR2* variants can lead to arrhythmias is important as treatment efficacy may be dependent on the underlying mechanism which leads to arrhythmias.

We identified a novel nonsense variant in *RYR2* in a young woman who suffered an unexplained cardiac arrest. To understand whether this variant causes arrhythmias by a different mechanism to the majority of missense variants in *RYR2*, we studied cardiomyocytes differentiated from hiPSCs generated from the patient.

## 2 MATERIALS AND METHODS

For detailed methods see Supplementary Methods.

### 2.1 Generation and maintenance of human induced pluripotent stem cells and differentiation into cardiomyocytes

hiPSCs were generated by retroviral infection of dermal fibroblasts obtained from a young woman carrying a heterozygous nonsense variant in *RYR2*, p.(Arg4790Ter). The woman was 32 years old at the time of the skin biopsy collection.

The control cells used in this study were generated from a well‐established and previously characterized control hiPSC line (Maizels et al., 2017; Shinnawi et al., 2015) which was generated from an individual with no personal or family history of cardiac disease.

hiPSCs were cultured on 1:200 growth‐factor reduced Matrigel (Corning) in mTeSR1 culture medium (Stem Cell Technologies) with the medium being refreshed daily. Cardiomyocytes were generated from a monolayer of hiPSCs using a previously published chemically defined differentiation protocol (Burridge et al., 2014).

### 2.2 Quantitative reverse transcriptase polymerase reaction (RT‐PCR) and western blot

Total RNA and protein were extracted from nonsense variant carrying hiPSC‐CMs (RYR2‐hiPSC‐CMs) and control hiPSC‐CMs. Allele‐specific RT‐PCR was performed using primers designed on single nucleotide polymorphisms (SNPs) in *RYR2* (Supplementary Materials, Table S2).

Western blot analysis was performed to assess total RYR2 protein levels and determine whether the mutant RYR2 protein was present in RYR2‐hiPSC‐CMs. Two RYR2 antibodies were used; ARP106/1 (gift from Prof Williams' laboratory Swansea (West et al., 2002), 1:500) reacts to an epitope in the far C‐terminus (aa 4957–4967) which lies distal to the p.(Arg4790Ter) variant and sc‐376507 RYR2 antibody (Santa Cruz, 1:500) which reacts to an epitope in the N‐terminus, a region common to both the mutant and wild‐type RYR2. A ratio of expression of these was then calculated for the RYR2‐hiPSC‐CMs and compared to the expression ratio in the control hiPSC‐CMs.

Western blot analysis was also performed on RYR2‐hiPSC‐CMs transduced with an allele‐specific shRNA to assess total RYR2 protein levels. For this, the sc‐376507 RYR2 antibody (Santa Cruz, 1:500) was used.

### 2.3 Laser Confocal Ca2+ imaging

hiPSC‐CMs were enzymatically dissociated with TrypLE Express (Life Technologies) and plated onto Matrigel coated 35 mm glass bottom optical culture dishes (MatTek Corporation). 100,000–200,000 hiPSC‐CMs were plated onto the glass bottom of dish. Cells were loaded with 5 µM Fluo‐4AM (Molecular Probes). Calcium transients were recorded from spontaneously beating single hiPSC‐CMs using the line scan mode of a Zeiss LSM‐710 or LSM7 confocal system. All experiments were performed in tyrodes solution at 37°C.

hiPSC‐CMs were line scanned at baseline, 10 µM isoproterenol was applied and the cells were incubated with this for 20 min before the same cells were re‐scanned.

Store overload induced calcium release (SOICR) experiments were undertaken as previously described by Itzhaki et al (Itzhaki et al., 2012). In summary, hiPSC‐CMs were treated with tetrodotoxin (10 µM), lidocaine (50 µM) and cesium chloride (5 mM). hiPSC‐CMs were then exposed to external solutions of varying calcium concentrations. At each external calcium concentration, the same cells were scanned for 60 s using the line scan mode and spontaneous calcium releases (small irregular releases occurring in the absence of an action potential) were recorded.

For beta‐blocker experiments, hiPSC‐CMs were incubated with a beta‐blocker for 20 min and then scanned. The proportion of cells displaying abnormalities at baseline and after incubation with the beta‐blocker were compared.

### 2.4 shRNA design and generation

Allele‐specific short hairpin RNAs (shRNAs) to target the mutant *RYR2* allele were designed. The allele‐specificity was based on the nonsense variant and in each shRNA the mutation recognition site was located in a different position (Supplementary Materials, Table S3).

RYR2‐hiPSC‐CMs were lentivirally transduced with the shRNAs and RNA was subsequently extracted to perform allele‐specific RT‐PCR to assess the expression of the mutant and wild‐type alleles.

For calcium imaging, the shRNAs were cloned into a vector containing Ds‐Red, in order to identify effectively transduced cells. The transduced hiPSC‐CMs were loaded with Fluo‐4‐AM. Cells which had been successfully transduced, identified by DsRed, were line scanned.

### 2.5 Statistical analysis

Continuous variables were expressed as mean ± SEM and differences were assessed using the student*’*s *t*‐test. Categorical differences between groups were assessed using the chi‐squared test. A *p* value of less than 0.05 was deemed statistically significant.

## 3 RESULTS

### 3.1 Clinical details

**FIGURE 1:**
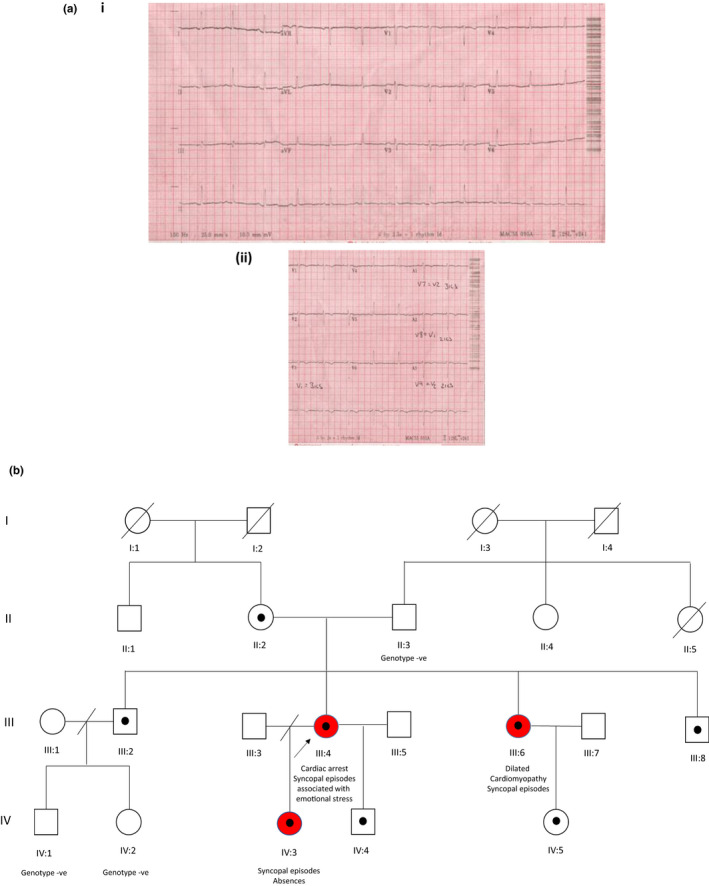
Clinical information regarding the proband and family in which the p.(Arg4790Ter) *RYR2* variant was identified. (a) Resting ECG traces from the proband. (i) resting 12 lead ECG shows no evidence of QT prolongation. (ii) ECG performed in the brugada position with extra precordial leads V3 = right parasternal 3rd intercostal space, A1 = Left parasternal 3rd intercostal space, A2 = Right parasternal 2nd intercostal space, A3 = Left parasternal 2nd intercostal space. The ECG shows no evidence of a Brugada pattern (ST elevation in the precordial leads). (b) Pedigree showing the family in which the p.(Arg4790Ter) *RYR2* variant was identified. Males are represented with squares and females with circles. The proband (III:4) is denoted with an arrow and individuals who were found to carry the p.(Arg4790Ter) variant are indicated with a black dot. Symptomatic individuals are shaded in red

The novel nonsense variant in *RYR2*, c.14368C>T, p.(Arg4790Ter), was identified in a woman in her fourth decade who suffered a cardiac arrest during sexual intercourse. Five years before this she suffered an episode of loss of consciousness during sexual intercourse. Following this initial episode, an MRI of the brain and an EEG were performed and both were reported as normal. During childhood, she suffered from numerous syncopal episodes associated with emotional stress. Investigations following the cardiac arrest revealed an unremarkable resting ECG with no evidence of a prolonged QT interval or a Brugada pattern (Figure 1(a)). A cardiac MRI revealed no structural cardiac abnormalities and left ventricular systolic function was reported as within normal limits. A coronary angiogram was normal. The patient suffered a hypoxic brain injury following her cardiac arrest and was unable to comply with an exercise stress test. A dual chamber ICD was implanted after her cardiac arrest and the patient is currently on bisoprolol (2.5 mg BD). Treatment with beta‐blockers has fully controlled the patient*’*s symptoms and no further arrhythmic episodes have been documented since her cardiac arrest nine years ago.

The proband initially underwent genetic testing using Next Generation Sequencing of a panel of 57 genes associated with inherited cardiac conditions (Manchester Regional Genetics Laboratory). This testing identified the p.(Arg4790Ter) variant in *RYR2*. The p.(Arg4790Ter) variant is not listed on dbSNP or gnomAD (n > 140,000) (Karczewski et al., 2020) as a rare polymorphism. Based on the ACMG guidelines the p.(Arg4790Ter) variant would be classified as a variant of uncertain significance (Richards et al., 2015). Most variants of *RYR2* may fall into this category as they are novel or very rare (Olubando et al., 2020). Although the variant is rare and is a nonsense variant, such variants in *RYR2* have not been reported as a disease mechanism. The p.(Arg4790Ter) variant was predicted to result in a truncated protein with a loss of 177 amino acids with a molecular weight of 544 kDa (compared to 565 kDa of wild‐type RYR2). Whole exome sequencing was undertaken on the proband*’*s DNA in order to rule out the presence of other variants which could be responsible for, or significantly contributing to, the phenotype. It is important to note that despite the patient suffering episodes of syncope associated with emotional stress and a cardiac arrest, polymorphic VT has never been documented which makes the phenotype of the patient atypical.

Following identification of the nonsense variant in the proband genetic testing was performed on other family members (Figure 1(b)). The proband*’*s mother (II:2) was found to carry the *RYR2* nonsense variant. She is in her 7th decade and has remained entirely asymptomatic. She has had a normal echocardiogram. Her exercise test was terminated following 5 min of the Bruce protocol due to dizziness however no arrhythmias were recorded. She has also had a 24 h ECG which showed sinus rhythm throughout with rare ectopics, including one triplet of atrial ectopics and a few ventricular ectopics. The proband has three siblings all of whom carry the nonsense variant. Both of her brothers (III:2 and III:8) have remained asymptomatic and have had normal cardiac investigations (echocardiograms and exercise tests). Her sister (III:6) suffered from syncopal episodes and had previously been given a diagnosis of epilepsy. She was also diagnosed with dilated cardiomyopathy during pregnancy. She has a normal resting ECG and exercise test. Her 24 h ECG showed sinus rhythm but some non‐conducted daytime P waves. The proband has two children (aged 12 and 17 years) both of whom carry the variant. The youngest (IV:4) is asymptomatic and has had normal cardiac investigations. The eldest (IV:3) has suffered from episodes of loss of consciousness and absences and, like her mother, initially underwent neurological investigation. She has a normal echocardiogram and resting ECG. A recent exercise test revealed increasing ectopy (including a triplet) at maximal exertion. Treatment with nadolol has improved her symptoms.

### 3.2 Characterization of calcium handling in the RYR2‐hiPSC‐CMs

Patient‐specific hiPSCs harboring the p.(Arg4790Ter) variant were generated. The presence of the nonsense variant was confirmed by sequencing gDNA (Supplementary Data, Figure S2). Karyotype analysis of the hiPSCs was regularly performed and both the control and the RYR2‐hiPSCs displayed normal karyotypes (Supplementary Data, Figure S3). The pluripotency of the hiPSCs was confirmed and the cells were successfully differentiated into spontaneously beating cardiomyocytes, RYR2‐hiPSC‐CMs (Supplementary Data, Figures S4–S6).

**FIGURE 2:**
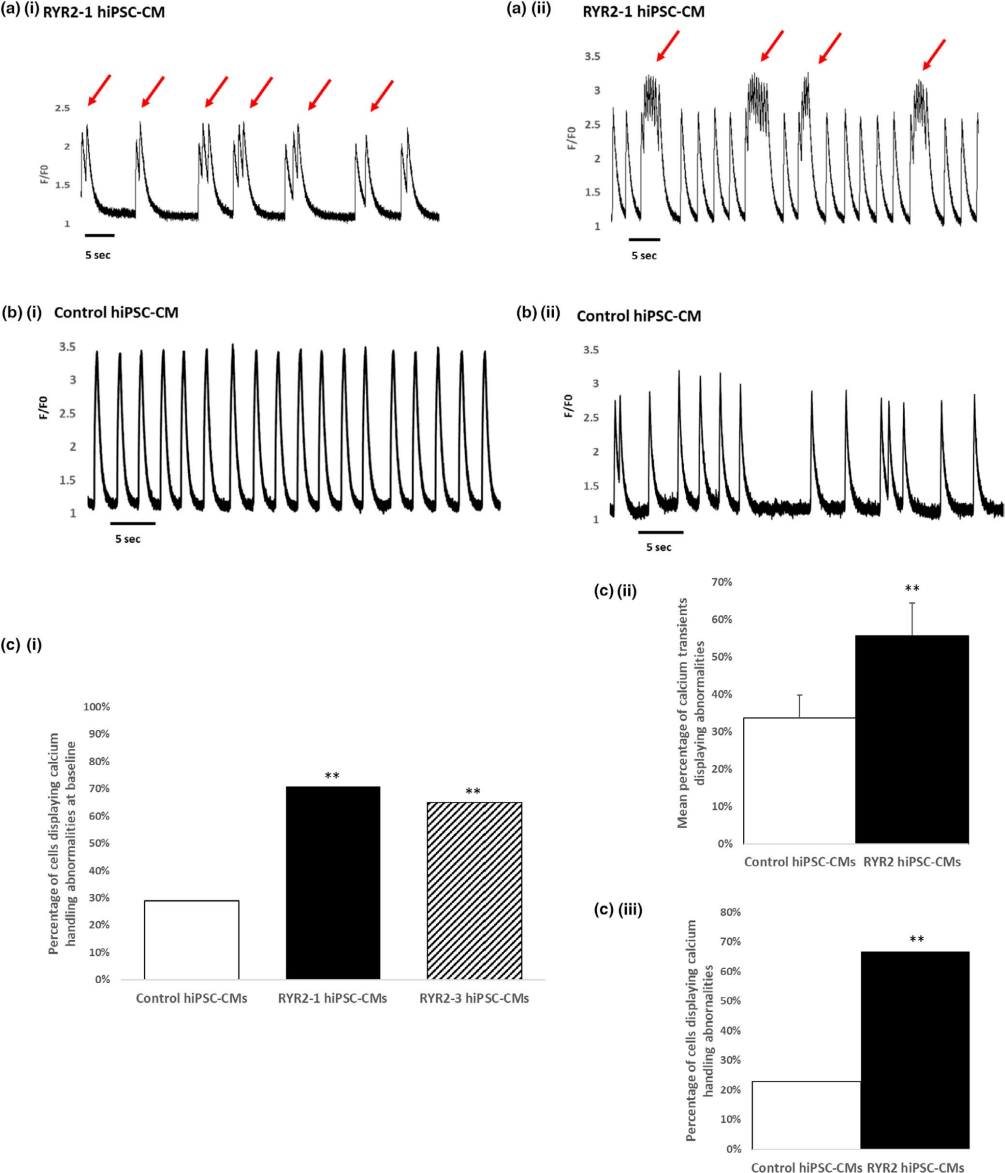
Calcium handling abnormalities in patient‐specific RYR2‐hiPSC‐CMs at baseline. (a) Line scan image showing changes in intracellular calcium in single RYR2‐hiPSC‐CMs at baseline illustrating the differences between double and triple peaked and broader multiple peaked transients. (a(i)) note the double and triple peaked transients (red arrows). (a(ii)) Note the broad multiple peaked transients (red arrows). (b) Line scan images showing changes in intracellular calcium in control hiPSC‐CMs at baseline. The abnormalities recorded in control hiPSC‐CMs tended to be less complex and less severe than those seen in RYR2‐hiPSC‐CMs. (c(i)) hiPSC‐CMs generated from both RYR2 clones (RYR2‐1 and RYR2‐3) displayed significantly more calcium handling abnormalities compared to healthy‐control hiPSC‐CMs (control 28.9% *n* = 121, RYR2‐1 70.9% *n* = 103, RYR2‐3 64.9% *n* = 259), ^**^ *p* < 0.001, chi‐squared test. (c(ii)) In cells displaying abnormalities, a higher percentage of calcium transients displaying abnormalities was observed in the RYR2‐hiPSc‐CMs compared to control hiPSC‐CMs (RYR2 55.74% *n* = 20, control 33.69% *n* = 12, ^**^ *p* < 0.001, chi‐squared test). (c(iii)) In cells displaying comparable spontaneous beating rates significantly more RYR2‐hiPSC‐CMs displayed calcium handling abnormalities compared to control hiPSC‐CMs (66.67% *n* = 30, control 22.86% *n* = 35, ^**^ *p* < 0.001, chi‐squared test, mean beat frequency 13.27/min and 14.14/min respectively)

Fluorescent calcium imaging revealed marked intracellular calcium transient abnormalities in the RYR2‐hiPSC‐CMs. These abnormalities were mainly large double and triple humped calcium transients (Figure 2(a) (i)), however, some were broader multiple peaked transients in which the peaks appeared less deep and narrower (A(ii)). The percentage of cells displaying calcium handling abnormalities was significantly higher in the hiPSC‐CMs derived from the two patient‐specific RYR2 hiPSC clones (70.9% and 64.9%) as compared to healthy‐control cells (28.9%, *p* < 0.001) (Figure 2c (i)). Moreover, although calcium handling abnormalities were observed in some of the control cells, these tended to be less complex than those seen in the RYR2‐hiPSC‐CMs (Figure 2(b)(ii)). There was no significant difference in the proportion of cells displaying abnormalities between cardiomyocytes generated from the two RYR2‐hiPSC clones. Looking at only cells which displayed abnormalities, the frequency of calcium transient abnormalities in each cell was quantified. This demonstrated that in RYR2‐hiPSC‐CMs the percentage of calcium transients affected by abnormalities was substantially higher than in control cells (55.74% vs. 33.69% *p* < 0.001) (Figure 2(c)(ii)). As only spontaneously beating hiPSC‐CMs were used the rate of beating varied between cells. Analysis of the proportion of cells displaying abnormalities was therefore performed in a subset of cells which displayed a similar rate (between 10 and 20 spontaneous transients per minute). In this group of cells, the RYR2‐hiPSC‐CMs displayed significantly more calcium handling abnormalities than the control hiPSC‐CMs (66.67% vs. 22.86%, *p* < 0.001, mean beat frequency 13.27/min and 14.14/min respectively, range 10–20/min) (Figure 2(c) (iii)).

#### 3.2.1 Adrenergic stimulation

**FIGURE 3:**
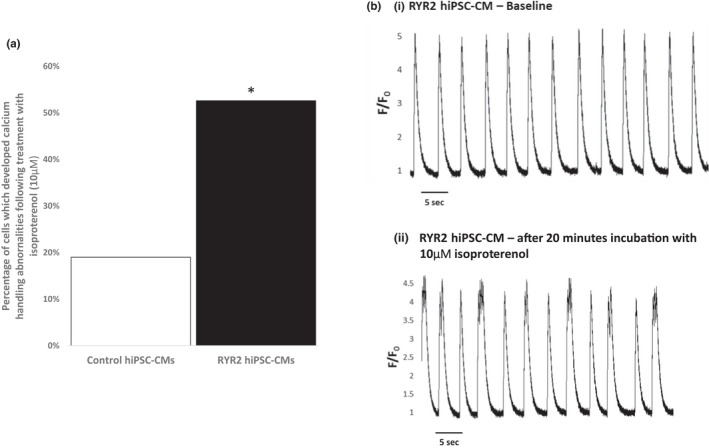
Calcium handling abnormalities observed in RYR2‐ and control hiPSC‐CMs following incubation with isoproterenol. (a) Significantly more RYR2‐hiPSC‐CMs which displayed normal transients at baseline developed calcium handling abnormalities following incubation with isoproterenol (10 µM) compared to control hiPSC‐CMs, 52.6% versus 19.0%, *n* = 19 and *n* = 21 for RYR2 and control cells respectively, ^*^ *p* < 0.05, chi‐squared test. (b) Whole cell calcium transients in a RYR2‐hiPSC‐CM at baseline (i) and after treatment with isoproterenol (ii). Note the development of double and triple humped transients after treatment with isoproterenol

As variants in *RYR2* are associated with adrenergically mediated arrhythmias, we tested the effect of adrenergic stimulation on the hiPSC‐CMs using isoproterenol. Focusing only on the hiPSC‐CMs that displayed normal calcium transients at baseline, 52.6% (10 of 19) of the RYR2‐hiPSC‐CMs developed calcium transient abnormalities after incubation with 10 µM isoproterenol, significantly more than seen in the healthy‐control cells (19.0%, *n* = 21, *p* < 0.05)—Figure 3(a). Cells typically developed multiple peaked transients after being incubated with isoproterenol (Figure 3(b)).

#### 3.2.2 Store overload‐induced calcium release

**FIGURE 4:**
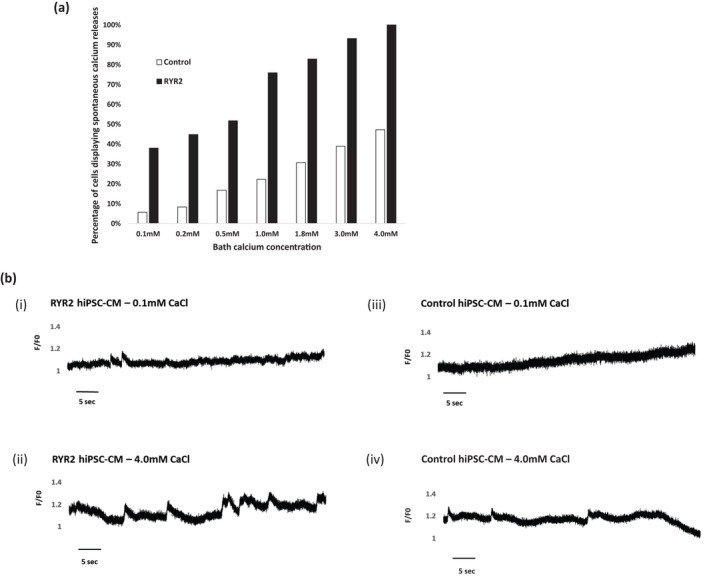
Effect of extracellular calcium concentration on calcium handling in RYR2‐ and control hiPSC‐CMs. (a) Percentage of RYR2‐hiPSC‐CMs (black) and healthy‐control hiPSC‐CMs (white) displaying spontaneous calcium transients and small calcium releases when bathed in solutions with varying calcium concentrations. *n* = 29 and *n* = 36 for RYR2 and control hiPSC‐CMs respectively, ^**^ *p* < 0.001 for all bath calcium concentrations, chi‐squared test. (b) Representative whole cell calcium traces from RYR2‐ and control hiPSC‐CMs at 0.1 mM and 4.0 mM

The p.(Arg4790Ter) variant is predicted to result in a truncated protein with the loss of a region which has been implicated in luminal calcium sensing (Chen et al., 2014). Increasing the sensitivity of ryanodine receptors to luminal calcium facilitates the development of spontaneous calcium release. We therefore, sought to assess whether the p.(Arg4790Ter) variant increased the propensity to develop spontaneous calcium releases at varying external calcium concentrations, the store overload‐induced calcium release (SOICR) threshold. As demonstrated in Figure 4, SOICR events were noted in both RYR2‐ and healthy‐control hiPSC‐CMs, and their occurrence increased with elevated calcium concentration. Importantly, SOICR threshold was significantly reduced in the RYR2‐hiPSC‐CMs and the percentage of RYR2‐hiPSC‐CMs displaying spontaneous calcium releases was significantly higher than control hiPSC‐CMs for each calcium concentration (*p* < 0.001 for all calcium concentrations, *n* = 29 for RYR2 and *n* = 36 for control, Figure 4).

These experiments clearly demonstrate that the p.(Arg4790Ter) variant increases the propensity of hiPSC‐CMs to develop spontaneous calcium releases and suggests that the nonsense variant causes a gain of function phenotype.

### 3.3 Drug screening in the RYR2‐hi PSC‐CMs

**FIGURE 5:**
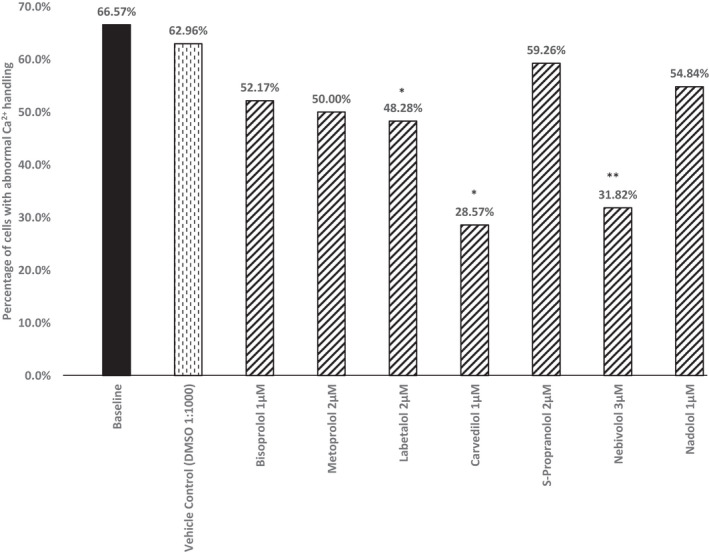
Effect of different beta‐blockers on the proportion of RYR2‐hiPSC‐CMs displaying calcium handling abnormalities. Treatment with 2 µM labetalol, 1 µM carvedilol and 1 µM nebivolol led to significant reductions in the proportion of cells displaying calcium transient abnormalities (^*^ *p* < 0.05, ^**^ *p* < 0.001 respectively, chi‐squared test). Treatment with the other beta‐blockers tested resulted in no significant difference. Baseline *n* = 362, vehicle control *n* = 27, bisoprolol *n* = 23, metoprolol *n* = 28, labetalol *n* = 29, carvedilol *n* = 14, S‐propranolol *n* = 27, nebivolol *n* = 22, nadolol *n* = 31

Both carvedilol and nebivolol inhibit ryanodine receptors (Tan et al., 2016; Zhou et al., 2011). To confirm that the abnormalities we detected in the RYR2‐hiPSC‐CMs are caused by a gain of function we tested the effects of these two beta‐blockers on the RYR2‐hiPSC‐CMs. To ensure the effects observed were not due to the beta‐blocker properties of these drugs, we performed these experiments in the absence of adrenergic stimulation and tested the effects of several other beta‐blockers as control. Treatment with both nebivolol and carvedilol resulted in significant reductions in the proportion of cells displaying calcium handling abnormalities (Figure 5). Treatment with labetalol also resulted in a small reduction in the proportion of hiPSC‐CMs displaying abnormal calcium handling.

### 3.4 The effect of the p.(Arg4790Ter) variant on the presence of the RYR2 protein

**FIGURE 6:**
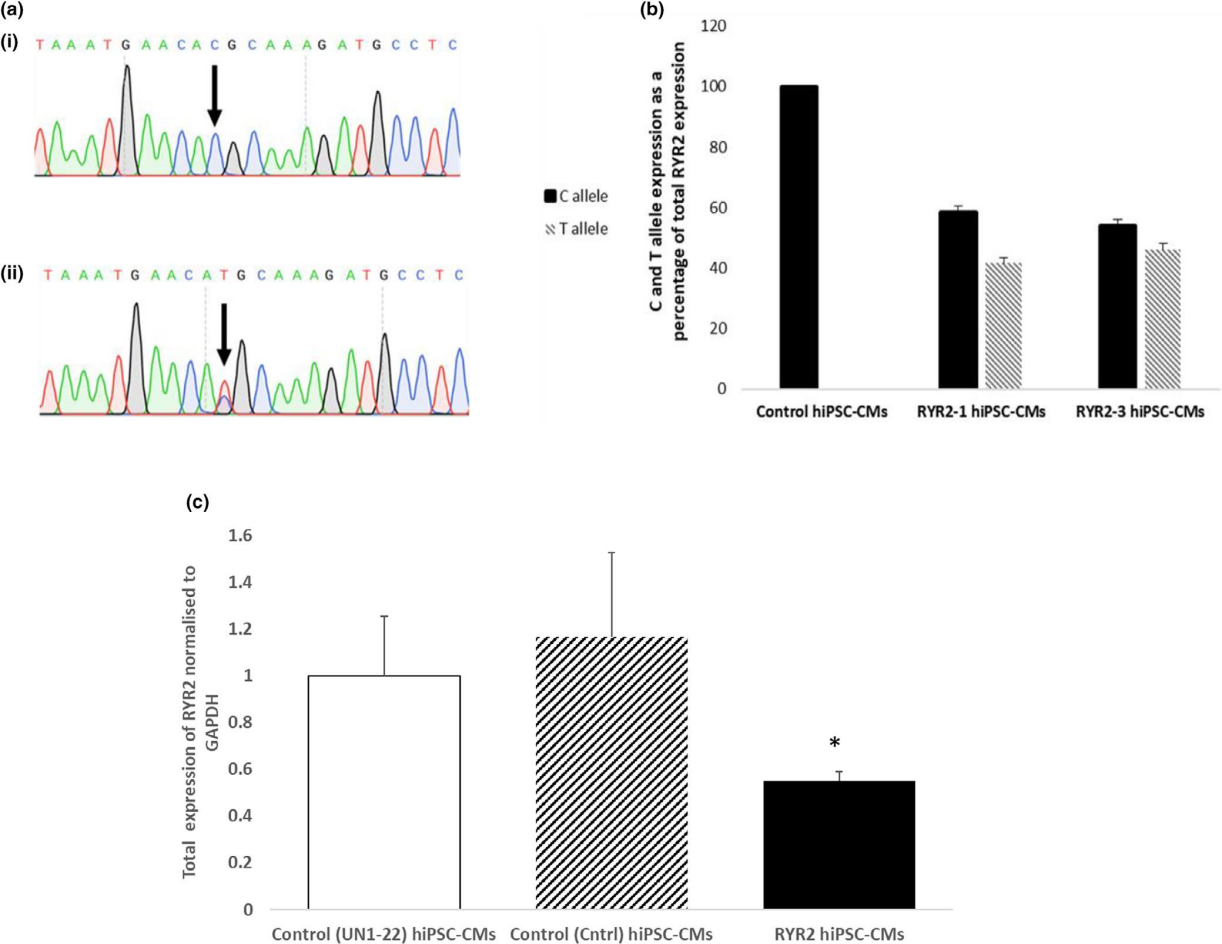
RYR2 expression in hiPSC‐CMs harbouring the nonsense variant. (a) Sequencing of DNA extracted from healthy‐control hiPSCs showed that the line is homozygous for the rs684923 SNP in *RYR2* ((i)) whilst the RYR2 line is heterozygous for the SNP ((ii)). (b) Allele‐specific RT‐PCR confirmed the expression of both RYR2 alleles in hiPSC‐CMs generated from both clones of the RYR2 line (RYR2‐1 and RYR2‐3). Only the C allele was detected in control hiPSC‐CMs confirming the specificity of the primers. (c) RT‐PCR for total *RYR2* expression performed on RNA extracted from hiPSC‐CMs derived from two different control lines (UN1‐22 and Ctrl) and the RYR2‐hiPSC‐CMs (*n* = 4, 2 and 5 respectively). In RYR2‐hiPSC‐CMs, total *RYR2* expression was significantly reduced compared to expression in the control lines,^*^ *p* < 0.05, student*’*s t‐test. There was no significant difference in *RYR2* expression between the two healthy‐control lines

To understand how the p.(Arg4790Ter) variant leads to a gain of function we designed an allele‐specific RT‐PCR based on a SNP (rs684923, c.7806C>T, MAF 45%) in *RYR2*, aiming to determine whether the mutant *RYR2* allele is expressed. Sequencing of gDNA confirmed that the RYR2‐hiPSC‐CMs were heterozygous for the SNP rs684923 whilst the control cells were homozygous (Figure 6(a)). The allele‐specific RT‐PCR showed expression of both alleles at the mRNA level in hiPSC‐CMs derived from two different RYR2‐hiPSC clones (RYR2‐1 and RYR2‐3) (Figure 6(b)). This finding suggests that the transcript escapes nonsense mediated decay. As expected, RT‐PCR only detected one allele in the control hiPSC‐CMs confirming the specificity of the primers. Despite the expression of both alleles in the RYR2‐hiPSC‐CMs, total *RYR2* expression was found to be significantly reduced in the RYR2‐hiPSC‐CMs compared to expression in control (UN1‐22) hiPSC‐CMs and also hiPSC‐CMs generated from another independent control line (Figure 6(c)). There was no significant difference in total RYR2 expression between hiPSC‐CMs derived from the two different healthy‐control lines.

**FIGURE 7:**
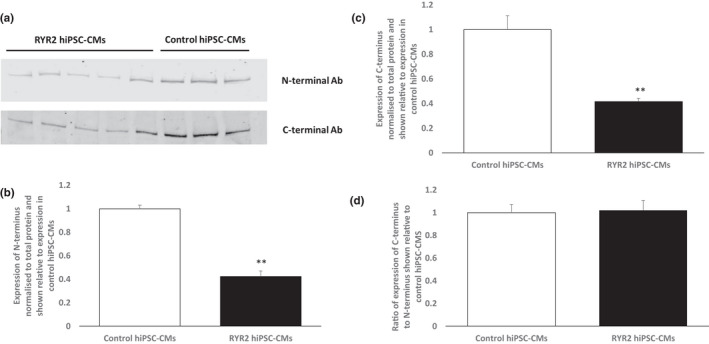
Western blot to assess RYR2 levels. (a) Representative western blots showing expression of N‐terminus and C‐terminus in protein extracted from RYR2‐hiPSC‐CMs and control hiPSC‐CMs. (b) Quantification of expression of N‐terminus (total) of RYR2 in RYR2 and control hiPSC‐CMs. Data normalized to total protein. Total RYR2 expression was significantly reduced in the RYR2‐hiPSC‐CMs compared to the control hiPSC‐CMs, ^***^ *p* < 0.0005, student*’*s t‐test. (c) Quantification of expression of C‐terminus of RYR2 in RYR2‐ and control hiPSC‐CMs. Data normalized to total protein. Expression of the C‐terminus in RYR2 hiPSC‐CMs was significantly reduced compared to expression in control hiPSC‐CMs, ^**^ *p* < 0.001, student*’*s *t*‐test. (d) Ratio of expression of C‐terminus to N‐terminus in RYR2‐hiPSC‐CMs shown relative to expression in control hiPSC‐CMs. No significant difference was seen between the RYR2‐ and control hiPSC‐CMs. *n* = 5 (RYR2) and *n* = 3 (control)

Western blotting showed a significant reduction in total RYR2 protein levels in the RYR2‐hiPSC‐CMs compared to control (Figure 7(b) and Supplementary Data, Figure S7). There was no significant difference in the ratio of expression of the N‐terminus to C‐terminus in the RYR2‐hiPSC‐CMs compared to control (Figure 7(d)). An elevated N‐terminus to C‐terminus ratio in the RYR2‐hiPSC‐CMs compared to control would be expected if the truncated protein is present. These data suggest that the truncated RYR2 is not present.

### 3.5 Partial silencing of the RYR2 allele harboring the nonsense variant reduces calcium handling abnormalities

**FIGURE 8:**
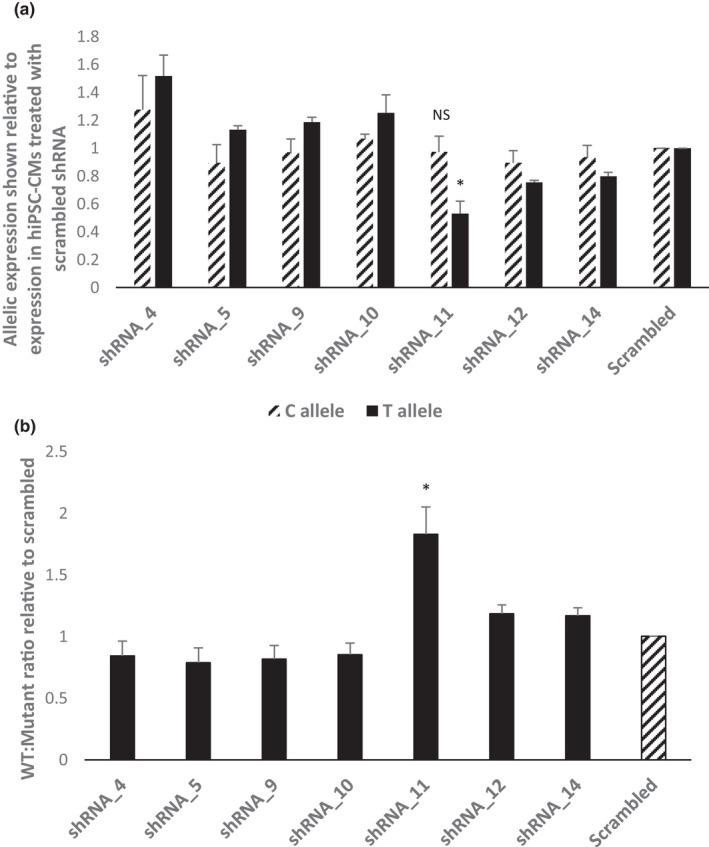
Allele‐specific RT‐PCR based on rs684923 performed on RNA extracted from RYR2‐hiPSC‐CMs treated with allele‐specific shRNAs and a scrambled shRNA. (a) Allelic expression was normalized to total *RYR2* expression and is shown relative to the expression in the hiPSC‐CMs treated with the scrambled shRNA. shRNA_11 led to a significant reduction in expression of the mutant allele (T allele) compared to treatment with the scrambled shRNA (^*^ *p* < 0.05, student*’*s *t*‐test). There was no significant difference (NS) between expression of the wild‐type (C allele) in the hiPSC‐CMs treated with shRNA_11 compared to those treated with the scrambled shRNA. *n* = 3 independent experiments. (b) The wild‐type to mutant *RYR2* allelic ratio in hiPSC‐CMs treated with allele‐specific shRNAs, shown relative to scrambled. shRNA_11 results in the largest increase in wild‐type:mutant ratio (^*^ *p* < 0.05, student*’*s *t*‐test). *n* = 3 independent experiments

In order to ascertain whether the abnormal calcium handling phenotype seen in the RYR2‐hiPSC‐CMs is due to an effect caused by the presence of the p.(Arg4790Ter) RYR2 allele, allele‐specific shRNAs were used to silence the mutant allele. The effect of seven different *RYR2* allele‐specific shRNAs on the expression of the mutant *RYR2* allele in the RYR2‐hiPSC‐CMs was assessed. RT‐PCR using allele‐specific primers based on the rs684923 SNP in *RYR2* was performed. Segregation studies performed on family members of the proband identified that the T allele segregated with the nonsense variant and could be used to reflect the expression of the mutant allele (Supplementary Data, Figure S8). shRNA_11 (in which the mutation recognition site was located at position 11 from the 5’ end of the shRNA) resulted in a significant reduction in expression of the mutant allele (Figure 8(a)) and a significant increase in the wild‐type/mutant allele expression (Figure 8(b)).

**FIGURE 9:**
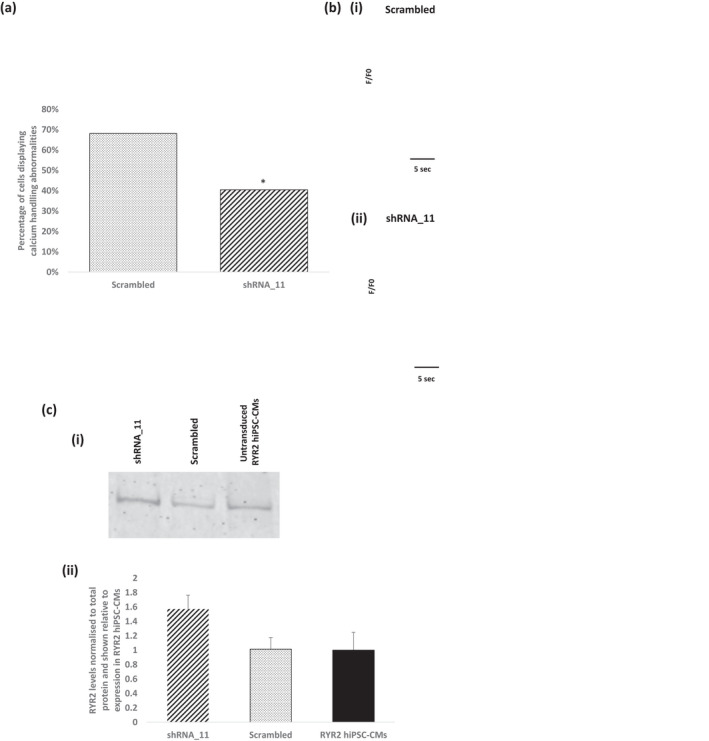
The effect of treatment with shRNA_11 on calcium handling and RYR2 protein levels in RYR2‐hiPSC‐CMs. (a) Treatment with shRNA_11 led to a significant reduction in the proportion of cells displaying abnormalities of the calcium transient compared to those treated with the scrambled shRNA. 68.2% of cells treated with scrambled (*n* = 22) and 40.5% of cells treated with shRNA_11 (*n* = 27) displayed calcium transient abnormalities (^*^ *p* < 0.05, chi‐squared test). (b) (i) Line scan image showing changes in intracellular calcium in a single RYR2‐hiPSC‐CM treated with the scrambled shRNA. (ii) Line scan image showing changes in intracellular calcium in a single RYR2‐hiPSC‐CM treated with shRNA_11. (c) The effect of treatment with shRNA_11 on total RYR2 protein levels. (i) Representative western blot showing total RYR2 expression in RYR2‐hiPSC‐CMs transduced with shRNA_11 and a scrambled shRNA and also untransduced RYR2‐hiPSC‐CMs. (ii) Treatment with shRNA_11 resulted in an increase in total RYR2 protein levels compared to those treated with the scrambled shRNA and the untransduced cells however this was not statistically significant. *n* = 3 each group

To determine the functional outcome of partial silencing of the mutated allele, we repeated the calcium imaging experiments on the RYR2‐hiPSC‐CMs transduced with allele‐specific shRNA_11. As shown in the representative calcium transients and in the quantitative summary of the percentage of cells displaying calcium transient abnormalities (Figure 9), shRNA_11 treated RYR2‐hiPSC‐CMs displayed a significant reduction in calcium handling abnormalities compared to cells treated with the scrambled shRNA (40.5% vs. 68.2%).

Western blotting performed on protein extracted from RYR2‐hiPSC‐CMs transduced with shRNA_11, RYR2‐hiPSC‐CMs transduced with a scrambled shRNA and also untransduced RYR2 hiPSC‐CMs showed increased levels of total RYR2 in the cells treated with shRNA_11 compared to those treated with the scrambled shRNA and the untransduced cells, however this was not statistically significant, *p* = 0.09 (Figure 9(b) and Supplementary Data, Figure S7).

## 4 DISCUSSION

In this study, we have used hiPSC‐CMs to elucidate the arrhythmogenic mechanism of a nonsense variant of the *RYR2* gene detected in a young patient who suffered a cardiac arrest. To the best of our knowledge, this is the first described nonsense variant of *RYR2* associated with an arrhythmogenic phenotype. We have made two important observations; (1) The nonsense variant causes profound alterations in calcium handling that are likely to cause arrhythmias (2) These alterations are due to a gain of function of the cardiac ryanodine receptor and are abolished by inhibitors of RYR2.

### 4.1 The p.(Arg4790Ter) variant causes profound calcium handling alterations that are likely to cause arrhythmias

The RYR2‐hiPSC‐CMs displayed significant calcium handling abnormalities characterized by prolonged transients with multiple peaks. These abnormalities can be triggered by isoproterenol. Similar abnormalities of the calcium transient have been previously reported in hiPSC‐CMs derived from patients with CPVT caused by *RYR2* variants (Itzhaki et al., 2012; Kujala et al., 2012; Novak et al., 2015). In some of these studies, it has been shown that these abnormalities lead to the formation of delayed afterdepolarizations (DADs) and Phase 3 early afterdepolarizations (Itzhaki et al., 2012; Kujala et al., 2012). Although the effect of the calcium handling abnormalities observed in the RYR2‐hiPSC‐CMs on the action potential was not assessed it is reasonable to hypothesize that the same mechanism occurs.

Although the presence of the truncated protein was not confirmed, treatment with the allele‐specific shRNA, which caused a reduction in the expression of the mutant allele, resulted in a reduction in calcium handling abnormalities supporting the conclusion that expression of the p.(Arg4790Ter) allele is important in the development of the arrhythmogenic phenotype.

### 4.2 Are the calcium handling abnormalities produced by the p.(Arg4790Ter) RYR2 variant due to a gain or a loss of function?

Most variants of *RYR2* known to cause CPVT result in a gain of function causing an increase in channel sensitivity to calcium facilitating the onset of spontaneous calcium release and DADs following adrenergic stimulation. It has been suggested that some *RYR2* variants can result in a loss of function due to a decreased sensitivity of the channel to activation by calcium (Jiang et al., 2007; Zhao et al., 2015). Loss of function leads to an excessive accumulation of calcium in the sarcoplasmic reticulum that predisposes to early afterdepolarizations (Zhao et al., 2015).

Three main lines of evidence support the conclusion that the p.(Arg4790Ter) variant acts in gain of function; (1) The calcium handling abnormalities observed in the RYR2‐hiPSC‐CMs at baseline were largely multiple peaked transients. These abnormalities have previously been reported in hiPSC‐CMs harboring gain of function variants of *RYR2* (Itzhaki et al., 2012; Novak et al., 2015). These abnormalities may represent calcium waves occurring during the calcium transient whilst the broader multiple peaked transients could potentially represent multiple calcium sparks. The RYR2‐hiPSC‐CMs also developed calcium handling abnormalities in response to treatment with isoproterenol. (2) The RYR2‐hiPSC‐CMs also displayed an increased tendency to develop spontaneous calcium release at various external calcium concentrations in comparison to the control hiPSC‐CMs. This suggests a reduced threshold for SOICR, a key characteristic of gain of function *RYR2* variants. (3) The calcium handling abnormalities were largely abolished by treatment with nebivolol or carvedilol. Treatment with labetalol was also shown to result in a reduction in calcium handling abnormalities in the RYR2‐hiPSC‐CMs. Both carvedilol and labetalol have alpha‐blocking properties. Interestingly, previous work has shown that alpha blockade potentiates CPVT therapy in calsequestrin mice (Kurtzwald‐Josefson et al., 2014). The experiments with the RYR2‐hiPSC‐CMs were, however, conducted in the absence of an adrenergic agonist, therefore it is unlikely that the effects observed with carvedilol and labetalol are due to their alpha blocking properties. Both carvedilol and nebivolol have previously been shown to inhibit and reduce the opening of ryanodine receptors. Both of these drugs have antioxidant properties and therefore it is not possible to determine whether the ability of carvedilol and nebivolol to reduce calcium handling abnormalities is purely due to the inhibition of ryanodine receptors. Antioxidant treatment has, however, been shown to inhibit ryanodine receptors (Terentyev et al., 2008). This suggests that, if there is a contribution of the antioxidant effect of these drugs, the underlying mechanism resulting in the changes in calcium handling are likely to be due to inhibition of ryanodine receptors.

### 4.3 How does the p.(Arg4790Ter) variant lead to a gain of function?

The RT‐PCR clearly demonstrated that the p.(Arg4790Ter) *RYR2* variant is expressed at mRNA level and escapes nonsense mediated decay. However, western blotting failed to confirm the presence of a truncated protein. A significant reduction in total RYR2 protein levels was observed. On the basis of these data, we are not able to explain how the p.(Arg4790Ter) *RYR2* variant leads to a gain of function. We can only speculate and provide two hypotheses; (1) The mutant protein is undetectable but present at a level sufficient to disrupt channel gating and calcium handling; (2) The substantial reduction in total RYR2 levels caused by the variant results in remodeling of the calcium handling machinery that leads to a gain of function. Tamoxifen inducible tissue specific *RYR2* knockout mice displaying a 50% reduction in RYR2 protein levels have been shown to display lower resting heart rates and episodes of tachyarrhythmia associated with stress (Bround et al., 2012). Further work has shown that a 50% reduction in RYR2 protein levels in mice results in alterations in the frequency and amplitude of calcium transients (Bround et al., 2016). This suggests that a reduction in RYR2 alone is sufficient to result in abnormal calcium handling and arrhythmias.

It is unclear how sensitive the western blotting approach using two different RYR2 antibodies to assess the presence of the mutant RYR2 protein is and whether it is able to detect low levels of the truncated protein. If translated, the truncated protein resulting from the p.(Arg4790Ter) variant would be missing part of the calcium‐sensing gate. It is plausible that the incorporation of one truncated monomer into the channel could lead to significant alterations in calcium handling. In view of this, it is reasonable to hypothesize that a low level of mutant protein could be sufficient to produce substantial disruption of calcium handling. Abnormal gating of half to a third of the total RYR2 tetramers would be sufficient to induce significant disruption of calcium handling similar to that observed in the RYR2‐hiPSC‐CMs. If we assume that between a third and a half of all RYR2 tetramers contain one truncated monomer the truncated monomers would represent between 8 and 12.5% of total RYR2. It is unclear whether the approach we utilized would enable us to detect these levels of expression.

Our data suggest that both *RYR2* alleles are expressed at mRNA level. However, the RYR2‐hiPSC‐CMs displayed a significant reduction in total RYR2 compared to the control hiPSC‐CMs at both mRNA and protein levels. To ascertain whether this could be due to cell line variation, the expression of total *RYR2* was assessed in hiPSC‐CMs derived from another independent control line. The expression of total *RYR2* in these control hiPSC‐CMs was not significantly different from the expression in the control cells used in this study. It is possible that the variant leads to reduced RYR2 levels and the loss of RYR2 initiates a complex remodeling of the calcium handling machinery that leads to an overall gain of function. This is of particular interest if we consider that the *RYR2* gene is highly intolerant of haploinsufficiency. In the gnomAD database, *RYR2* has a probability of loss of intolerance (pLI) of 1.0 (Karczewski et al., 2020).

Western blotting performed on samples from cells transduced with shRNA_11 showed increased levels of RYR2 protein compared to those transduced with the scrambled shRNA and also untransduced cells. This difference was not statistically significant, therefore, it is not possible to draw any definite conclusions from this data. However, if the mutant protein is synthesized but is cleared by quality control systems this could result in increased RYR2 protein levels in the cells transduced with shRNA_11 as in these cells there may be an increased wild‐type to mutant ratio at the protein level meaning a smaller proportion of RYR2 is cleared from the cell and therefore increased levels of RYR2 protein are present. It is possible mutant subunits are incorporated into tetramers but those containing a high number of mutant subunits are unstable and form aggregates or are removed from the cell. Further work is needed to assess the effects of reduction of RYR2 levels.

### 4.4 Limitations

It is well understood that hiPSC‐CMs do not display the structural organization seen in adult human cardiomyocytes however recently protocols have been developed to circumvent these issues including the development of T‐tubules which allow the close apposition of L‐type calcium channels with ryanodine receptors (Parikh et al., 2017). Although there has been some data to suggest that hiPSC‐CMs over 21 days of age display a robust calcium handling phenotype (Hwang et al., 2015), clearly the development of a more mature structural phenotype would improve this. The hiPSC‐CMs used in this study were all over 25 days old and it is possible that some of the calcium handling abnormalities observed at baseline may have been due to a degree of immaturity, however, abnormalities due to this would be expected to be comparable between the RYR2 and control hiPSC‐CMs.

In this study, we have not assessed whether the p.(Arg4790Ter) has any impact on the expression or function of other calcium handling proteins. However, despite this, the work undertaken provides initial interesting insights into how this nonsense variant leads to a phenotype.

One significant limitation of this study is the lack of an isogenic control line. This would have allowed the observed differences to be more directly attributed to the variant being studied. However, despite this, the work provides further insight into the mechanisms by which *RYR2* variants can lead to arrhythmias and also again highlights that hiPSC‐CMs can be used as an effective tool to provide insights into disease mechanisms and testing potential therapeutic agents.

## 5 CONCLUSION

The p.(Arg4790Ter) variant appears to behave like other gain of function variants in *RYR2*. The calcium handling abnormalities observed in the RYR2‐hiPSC‐CMs were improved by treatment with carvedilol and nebivolol suggesting these may be effective treatments for patients with gain of function variants in *RYR2*.

## ETHICS STATEMENT

The studies involving human participants were reviewed and approved by Central Manchester Research Ethics Committee. The patients/participants provided their written informed consent to participate in this study.

## AUTHOR CONTRIBUTIONS

William G Newman, Lior Gepstein, and Luigi Venetucci conceived the study. Benjamin Brown performed clinical evaluation of the proband. Irit Huber generated the hiPSC lines used in the study. Amira Gepstein, Gil Arbel and Claire Hopton cultured and differentiated hiPSCs. Leonid Maizels performed some SOICR experiments and Claire Hopton performed all other experiments. Claire Hopton, Luigi Venetucci and Anke J Tijsen interpreted results. Anke J Tijsen, Nicola Bates, Susan J Kimber, William G Newman, Luigi Venetucci, and Lior Gepstein provided supervision. Claire Hopton, William G Newman, Luigi Venetucci, and Lior Gepstein wrote the manuscript and all authors approved the final version.

## Supporting information



Fig S1‐S8

Supplementary Material
