# Erratum: Characterization of the mechanism by which a nonsense variant in *RYR2* leads to disordered calcium handling

**DOI:** 10.14814/phy2.15428

**Published:** 2022-08-08

The authors of the article by Claire Hopton et al. (2022) noticed an error in Figure 9b. The line scan traces showing changes in intracellular calcium are absent in Figure 9b.

The correct Figure 9b is as follows:

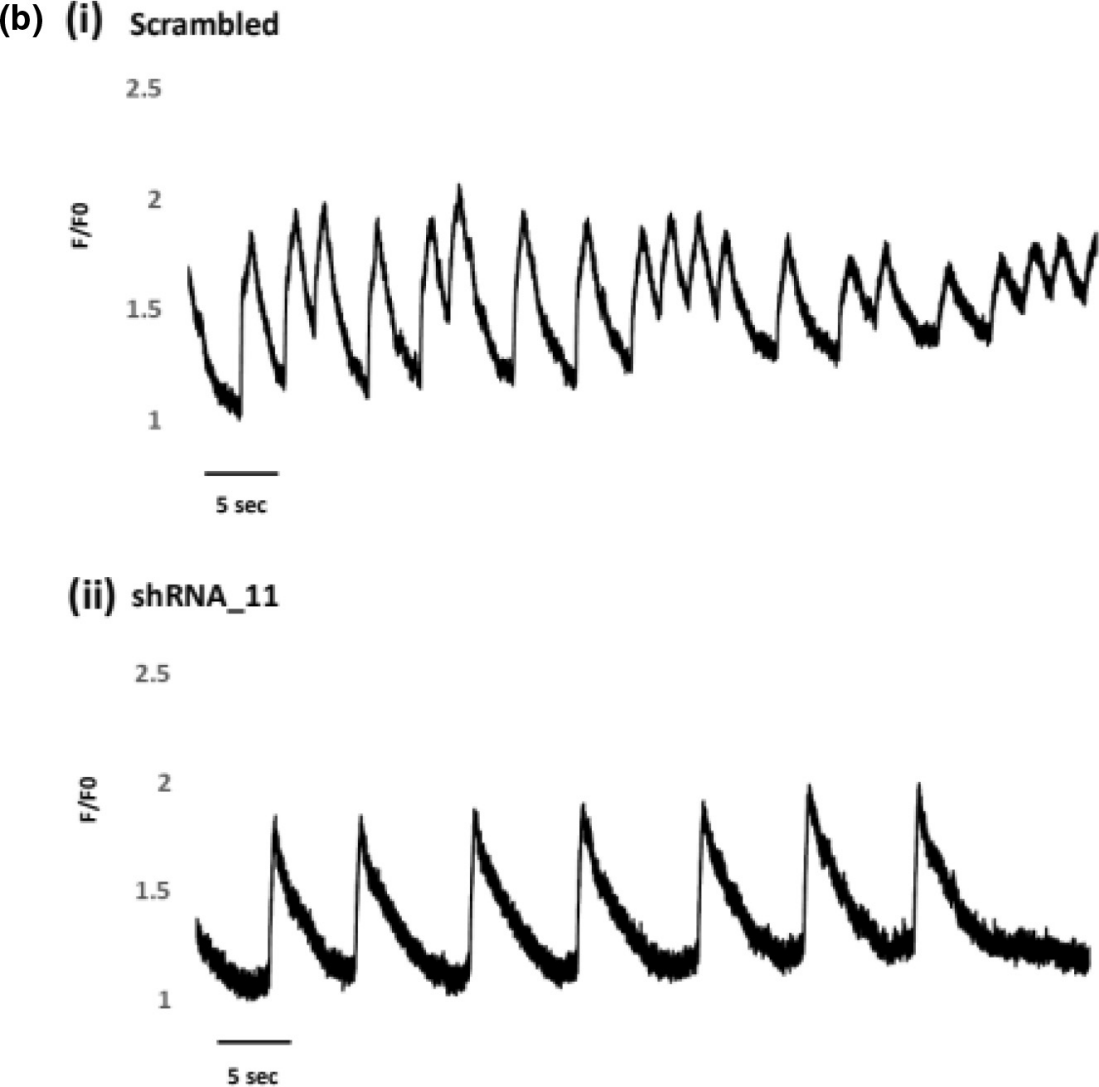

The publisher apologizes for this error.
