# Supplementary material for: Characterization of the mechanism by which a nonsense variant in RYR2 leads to disordered calcium handling
Source: Physiol Rep. 2022 Apr 19;10(8):e15265. doi: 10.14814/phy2.15265 (PMC9017975; doi:10.14814/phy2.15265)
Supplement: Supplementary file 1 — Fig S1‐S8 [file PHY2-10-e15265-s001.pdf]

Characterization of the mechanism by which a nonsense variant in *RYR2* leads to disordered calcium handling

Supplementary Data

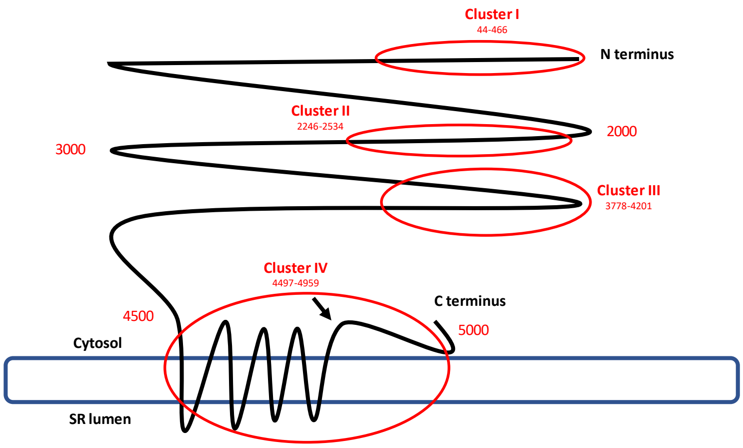

Figure S1. Schematic showing RYR2 protein. The four regions in which mutations are typically clustered are shown. The approximate location of the p.(Arg4790Ter) variant is shown with a black arrow. The variant results in the loss of 177 amino acids.

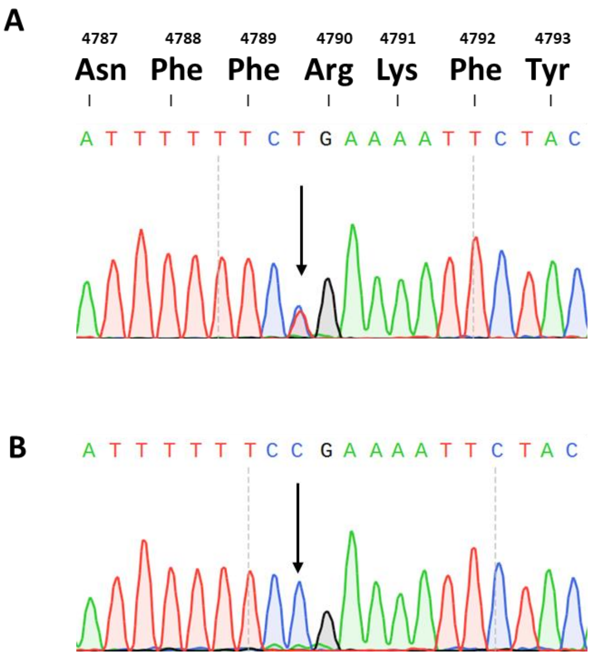

Figure S2. Sequencing of exon 100 in *RYR2* in RYR2 and control hiPSCs. (A) Sequencing of gDNA extracted from RYR2-1 hiPSCs confirmed the presence of the c.14368C>T p.(Arg4790Ter) variant. (B) Sequencing of gDNA from control hiPSCs confirmed the absence of this variant.

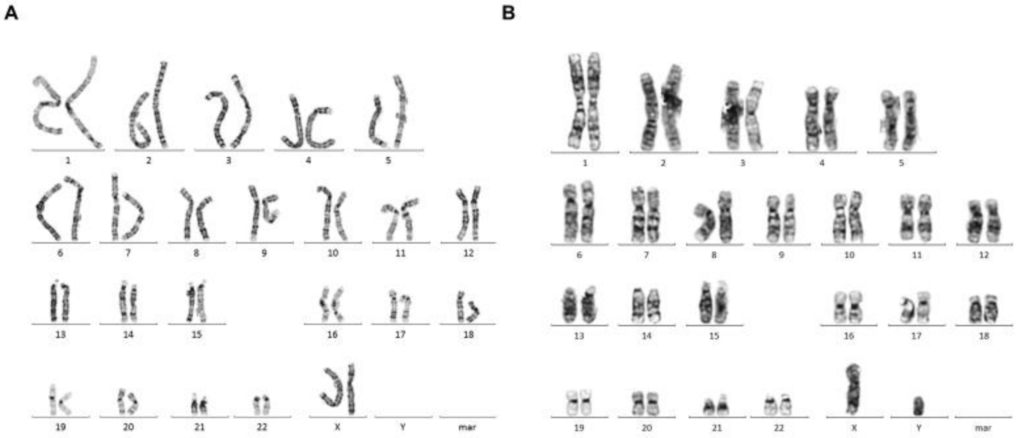

Figure S3. Karyotypes of hiPSCs. (A) Karyogram of RYR2-1 hiPSCs showing a normal female karyotype 46XX. (B) Karyogram of control hiPSCs showing a normal male karyotype, 46XY.

A

B

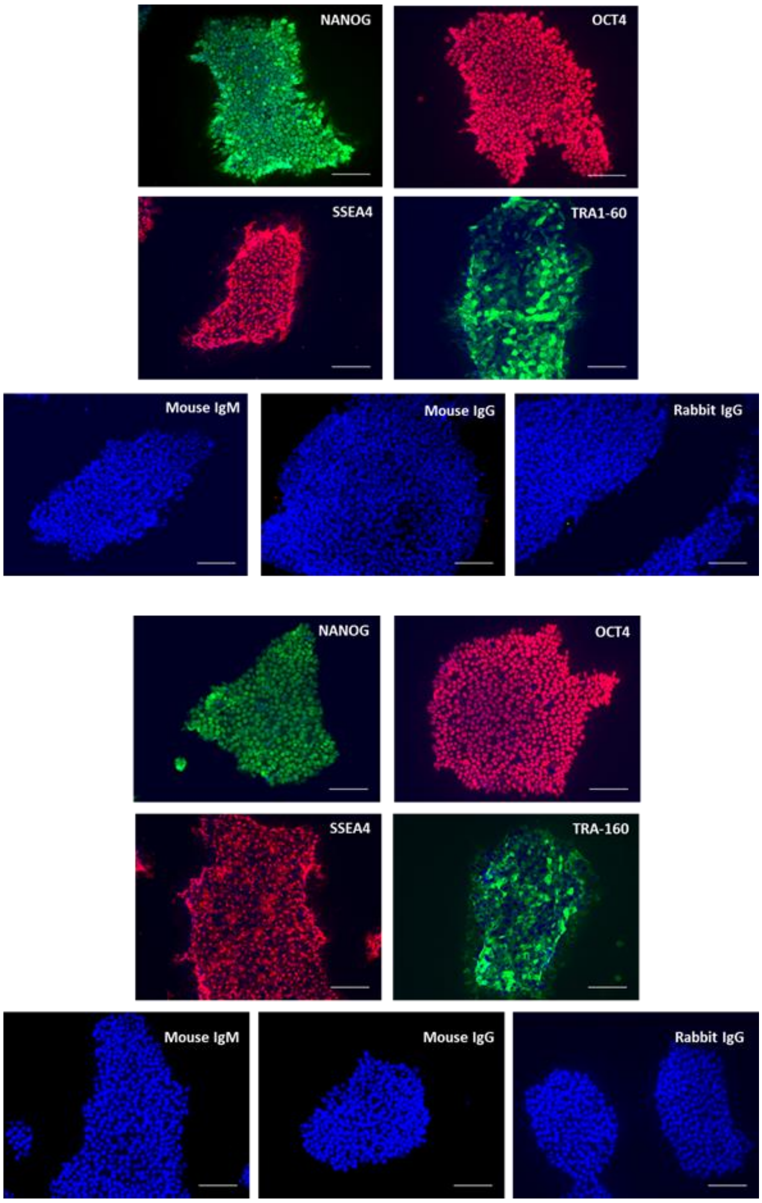

Figure S4. Immunostaining of RYR2 and control hiPSCs for pluripotency markers. Immunostaining of RYR2-1 (A) and control (B) hiPSCs for the pluripotency markers, Nanog, OCT4, SSEA4, TRA1-60 and also isotype controls (mouse IgM, mouse IgG and rabbit IgG). Nuclei stained with dapi (blue). Scale bars 100μm.

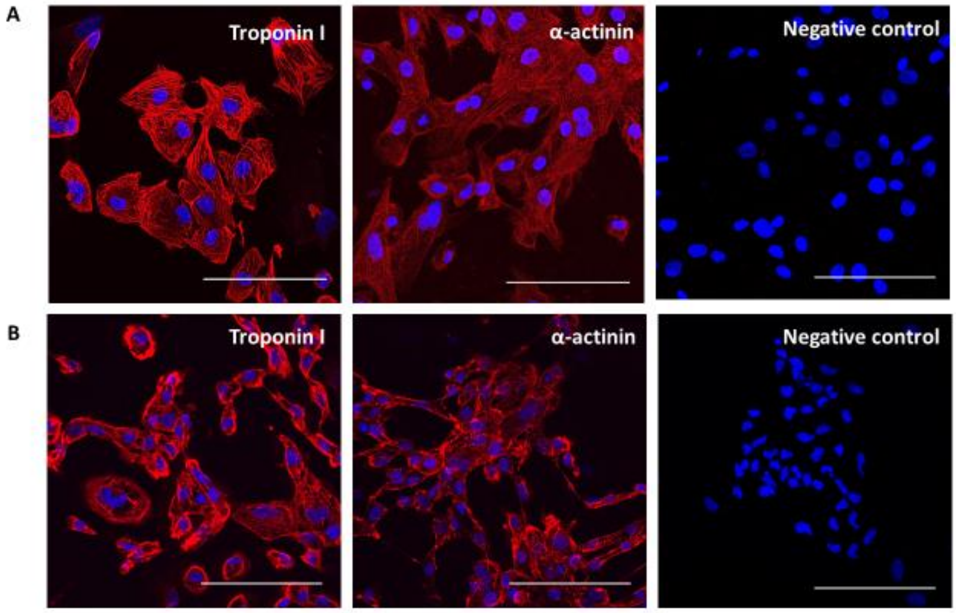

Figure S5. Immunostaining of RYR2 and control hiPSC-CMs for cardiac markers. Immunostaining of RYR2-1 (A) and control (B) hiPSC-CMs for the cardiac markers Troponin I (red) and α-actinin (red). Negative control (no primary antibody applied). Nuclei stained with dapi (blue). Scale bars 100µm.

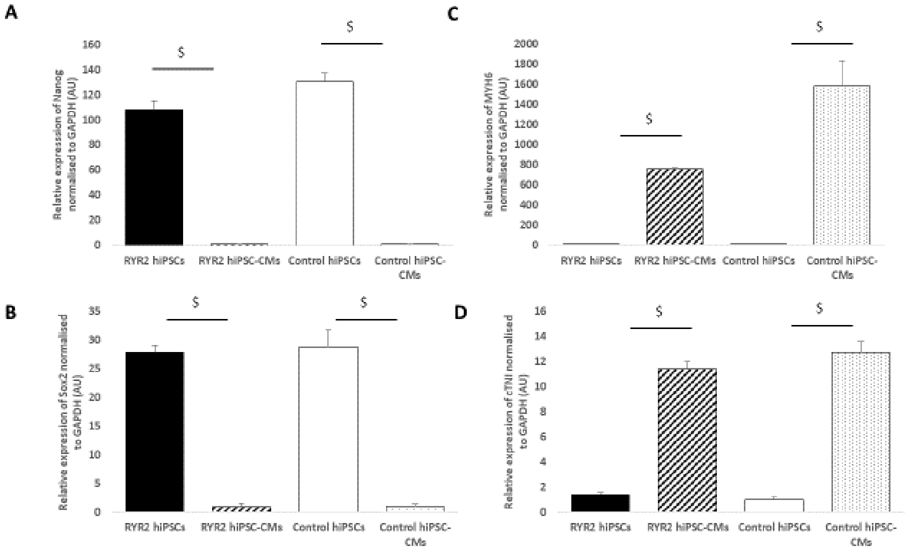

Figure S6. RT-PCR to assess expression of pluripotency and cardiac markers in hiPSCs and hiPSC-CMs. RT-PCR performed on cDNA synthesized from RNA extracted from RYR2-1 and control hiPSCs and hiPSC-CMs to assess the expression of Nanog (A), Sox2 (B), MYH6 (C) and cTNI (D) in RYR2 and control hiPSCs and hiPSC-CMs. Values normalised to GAPDH and expressed in arbitrary units (AU). Undifferentiated RYR2-1 and control hiPSCs displayed significantly higher expression of the pluripotency markers Nanog and Sox2 compared to the differentiated cells whilst the RYR2-1 and control hiPSC-CMs expressed significantly higher levels of the cardiac markers MYH6 and cTNI compared to the undifferentiated cells ($ p<0.0005, student's t-test). n=3 each group. Errors bars represent SEM.

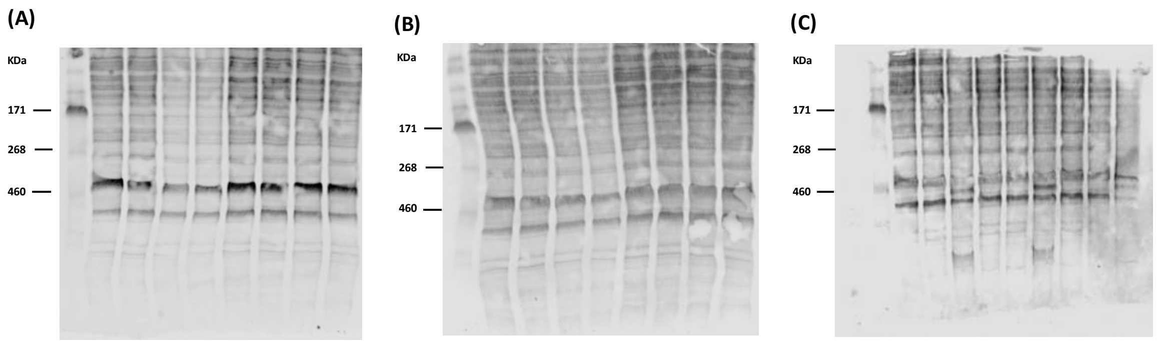

Figure S7. Western blot membranes stained for total protein. (A) Total protein stain on membrane used for N-terminal antibody to assess RYR2 protein levels in RYR2- and control-hiPSC-CMs. (B) Total protein stain on membrane used for C-terminal antibody to assess RYR2 protein levels in RYR2- and control-hiPSC-CMs. (C) Total protein stain on membrane used to assess total RYR2 protein levels in RYR2-hiPSC-CMs transduced with an allele-specific shRNA, RYR2-hiPSC-CMs transduced with a scrambled shRNA and untransduced RYR2-hiPSC-CMs

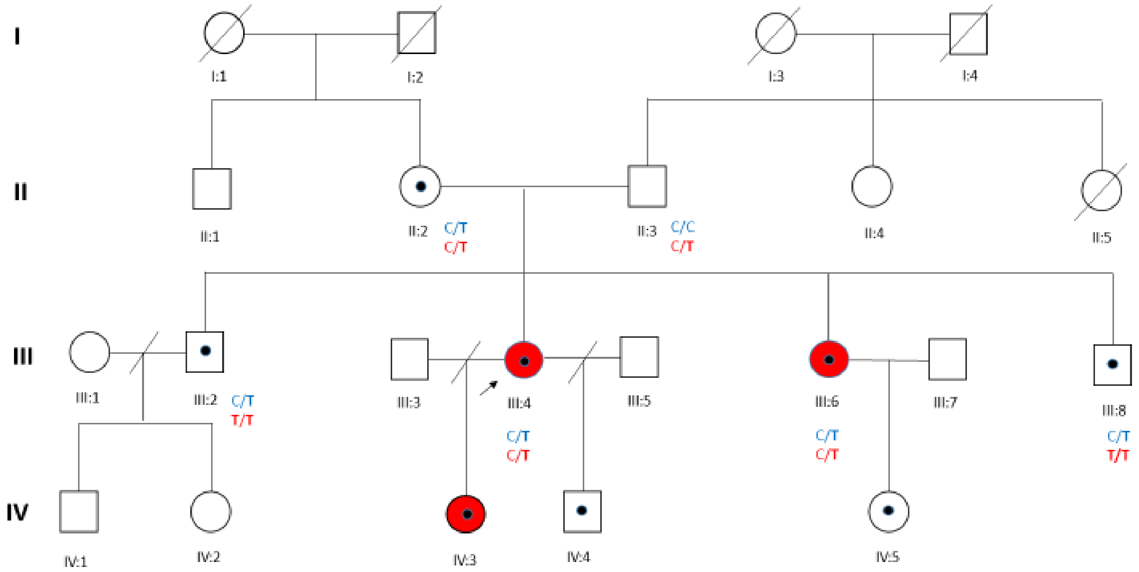

Figure S8. Pedigree in which the p.(Arg4790Ter) variant was identified showing genotyping for SNPs in *RYR2*. Males are represented with a square and females with a circle. The proband is indicated by an arrow and mutation carriers are indicated with a black dot. Individuals who are clinically symptomatic are shaded in red. Genotyping for the rs684923 (blue) and rs3765097 (red) shown below each individual with the variant allele being shown in bold.
